# Recombinant Encephalomyocarditis Viruses Elicit Neutralizing Antibodies against PRRSV and CSFV in Mice

**DOI:** 10.1371/journal.pone.0129729

**Published:** 2015-06-15

**Authors:** Shu Zhu, Xin Guo, Lisa R. Keyes, Hanchun Yang, Xinna Ge

**Affiliations:** 1 Key Laboratory of Animal Epidemiology and Zoonosis of Ministry of Agriculture, College of Veterinary Medicine and State Key Laboratory of Agrobiotechnology, China Agricultural University, Beijing 100193, People's Republic of China; 2 Department of Molecular Genetics and Microbiology, College of Medicine, University of Florida, Gainesville, Florida, United States of America; The University of Melbourne, AUSTRALIA

## Abstract

Encephalomyocarditis virus (EMCV) is capable of infecting a wide range of species and the infection can cause myocarditis and reproductive failure in pigs as well as febrile illness in human beings. In this study, we introduced the entire ORF5 of the porcine reproductive and respiratory syndrome virus (PRRSV) or the neutralization epitope regions in the E2 gene of the classical swine fever virus (CSFV), into the genome of a stably attenuated EMCV strain, T1100I. The resultant viable recombinant viruses, CvBJC3m/I-ΔGP5 and CvBJC3m/I-E2, respectively expressed partial PRRSV envelope protein GP5 or CSFV neutralization epitope A1A2 along with EMCV proteins. These heterologous proteins fused to the N-terminal of the nonstructural leader protein could be recognized by anti-GP5 or anti-E2 antibody. We also tested the immunogenicity of these fusion proteins by immunizing BALB/c mice with the recombinant viruses. The immunized animals elicited neutralizing antibodies against PRRSV and CSFV. Our results suggest that EMCV can be engineered as an expression vector and serve as a tool in the development of novel live vaccines in various animal species.

## Introduction

The manipulation of infectious cDNA clones has paved the way for using poliovirus as a vector to attempt the development of novel live recombinant vaccines [1–3]. Engineered chimeric polioviruses can express antigenic sites from different serotypes, other picornaviruses, or even heterologous pathogens such as human immunodeficiency virus (HIV). These recombinant viruses have been shown to be capable of eliciting specific neutralizing antibodies against the foreign determinants *in vivo* [4–7]. Subsequently, an attenuated Mengo virus strain has been selected as an alternative vector to poliovirus with the advantages including: i) the applicability in a wide host range, ii) incapability of integration into host genomes, iii) effective and stable expression of heterologous sequences and iv) no propensity for persistence subsequent to inoculation. Previous studies with the attenuated strain of Mengo virus has shown that it is able to present different foreign antigens fused into the viral leader protein (L protein) [8–10]. As another member of *Cardiovirus Genus*, Encephalomyocarditis Virus (EMCV) also has a 67-amino-acid leader protein highly homologous to Mengo virus. Hammouemi *et al* reported that they had successfully inserted enhanced green fluorescent protein (EGFP) into EMCV L protein and yielded recombinant virus progenies capable of producing fluorescence in the infected cells [11]. This observation implied that the EMCV L protein coding region is also competent in carrying foreign genes.

Encephalomyocarditis virus infects many mammals including rodents, livestock, wild animals, nonhuman primates and human beings [12–15]. Pigs are the most commonly and severely infected domestic animals whilst EMCV strains differ in their pathogenesis and tissue tropisms. For instance, G424/90 causes fatal myocarditis in piglets [16] whereas 2887A/91 mainly results in reproductive failure in sows [17]. Our previous investigation revealed that the EMCV seroprevalence in many intensive pig farms was as high as 53% in China, suggesting a wide spread of EMCV in the Chinese swine industry [18]. Regardless of the prevalence, most of these EMCV isolates exhibited asymptomatic infections, which may imply that strains emerging in China are less virulent than those isolated in the US and European countries [19]. EMCV BJC3, a Chinese isolate, was derived from an aborted swine fetus in 2004. We utilized this strain as a representative to study EMCV pathogenicity in mice and pigs. The data suggest that EMCV BJC3 had very mild or no clinical signs even in sucking piglets, although it resulted in a severe mortality in infected mice. In the previous study, we demonstrated that one single amino acid substitution (threonine to isoleucine) at position 100 of the capsid protein VP1 could remarkably attenuate the virus in mice [20]. The LD_50_ (50% Lethal Dose) of the less virulent virus, T1100I, was determined to be 5×10^5^ PFU when inoculated intraperitoneally and could be continuously propagated in BHK-21 cells without reversion. Given the fact that EMCV has been confirmed to be capable of accommodating EGFP in its L-protein coding region [11], we sought to insert our heterologous genes of interest into the EMCV backbone, which is attenuated enough to immunize mice and study the immunogenicity of these inserts *in vivo*. In this study, we engineered gene fragments either encoding the entire GP5 protein of the porcine reproductive and respiratory syndrome virus (PRRSV) strain JXwn06 or the A1A2 domain of the classical swine fever virus (CSFV) strain Shimen/HVRI glycoprotein E2 into the frame of EMCV strain T1100I L region. The recombinant viruses expressed the heterologous antigens in the form of fusion proteins, which can be recognized by either porcine anti-GP5 serum or anti-E2 monoclonal antibody. Furthermore, recombinant virus infection in BALB/c mice can induce neutralizing antibodies against PRRSV or CSFV, suggestive of the immunogenic competency of the expressed fusion proteins.

## Materials and Methods

### Ethics Statement

The mouse experimental procedures in this study were performed according to the Chinese Regulations of Laboratory Animals—The Guidelines for the Care of Laboratory Animals (Ministry of Science and Technology of People's Republic of China) and Laboratory Animal Requirements of Environment and Housing Facilities (GB 14925–2010, National Laboratory Animal Standardization Technical Committee). The license number associated with their research protocol was 20110611–01 and the animal study proposal was approved by The Laboratory Animal Ethical Committee of China Agricultural University.

### Construction of recombinant viruses

**Fig 1:**
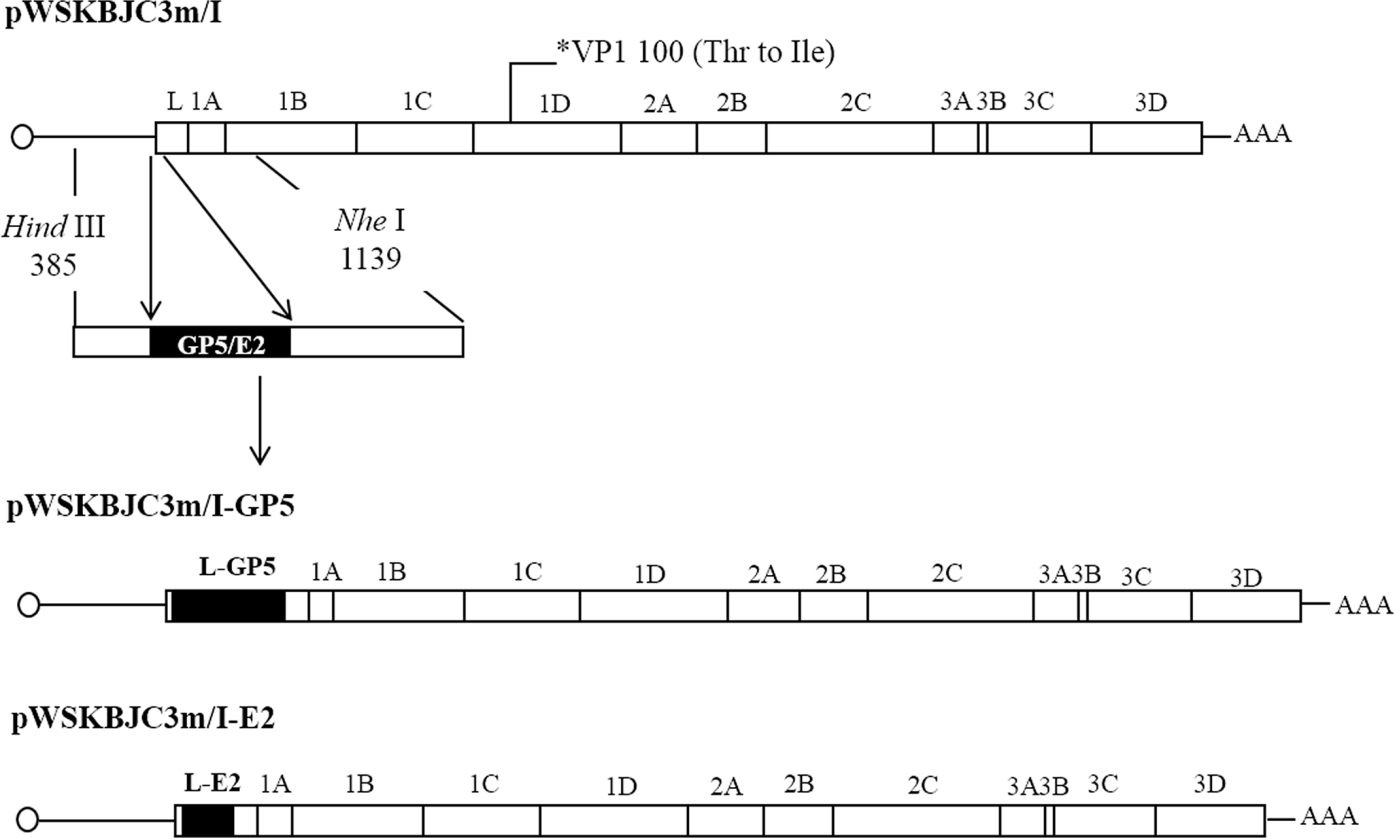
Schematic diagram of the construction of each recombinant virus full-length cDNA clone. The foreign sequences of PRRSV ORF5 and CSFV E2 neutralization epitopes A1A2 were amplified and fused into EMCV L sequences by fusion PCR. The fusion products were double digested with *Hind*Ⅲ and *Nhe*Ⅰ, gel purified and subcloned into pBluescript II SK^+^. Next, the gene fragments containing the heterologous genes were excised from the intermediate plasmids and cloned back to pWSKBJC3m/I to construct the full-length cDNA clones of recombinant viruses.

The construction of a full-length cDNA clone of the mutant EMCV strain T1100I, designated as pWSKBJC3m/I, was described previously [20]. We amplified the 630nt PRRSV JXwn06 ORF5 or a 204nt-length A1A2 fragment of CSFV Shimen/HVRI E2 gene from respective plasmid templates using primers that carry 5’ overhangs homologous to the desired insertion site in the L gene (S1 Table). The upstream and downstream fragments adjacent to the insertion point of EMCV L gene were also amplified and linked to PRRSV JXwn06 ORF5 or CSFV Shimen/HVRI E2 gene by fusion PCR. The resulting PCR product, which contained *Hind*Ⅲ and *Nhe*Ⅰ sites in the upper or lower homologous arms (Fig 1) were cloned into pBluescript II SK^+^ (Stratagene, Aarhus, Denmark) and confirmed by sequencing. These constructs were named pBLfGP5 and pBLfE2. For the construction of the recombinant virus full-length plasmids, pBLfGP5 and pBLfE2 were digested with *Hind*Ⅲ and *Nhe*Ⅰ, gel purified, and ligated to the *Hind*Ⅲ-*Nhe*Ⅰ digested pWSKBJC3m/I backbone. Two recombinant full-length cDNA clones were named pWSKBJC3m/I-GP5 and pWSKBJC3m/I-E2 (Fig 1).

The constructs were then linearized by *Bam*HⅠ and the infectious RNA transcripts were synthesized using T7 MEGAscript Kit (Ambion Inc., Texas, USA). BHK-21 cells were transfected with RNA transcripts as previously described [20]. The resultant recombinant EMCV chimeras were designated as CvBJC3m/I-ΔGP5 and CvBJC3m/I-E2. To generate the virus stocks, the transfected BHK-21 cellular lysates were propagated in BHK-21 cells for two additional passages and both cells and supernatants of Passage 3 (P3) were harvested when over 90% of the cells showed obvious cytopathic effect (CPE). The BHK-21 cell lysates were clarified by centrifugation at 3000 rpm for 15 min and 100μl aliquots were stored at -80°C.

### Stability and plaque morphology of the mutant viruses

**Fig 2:**
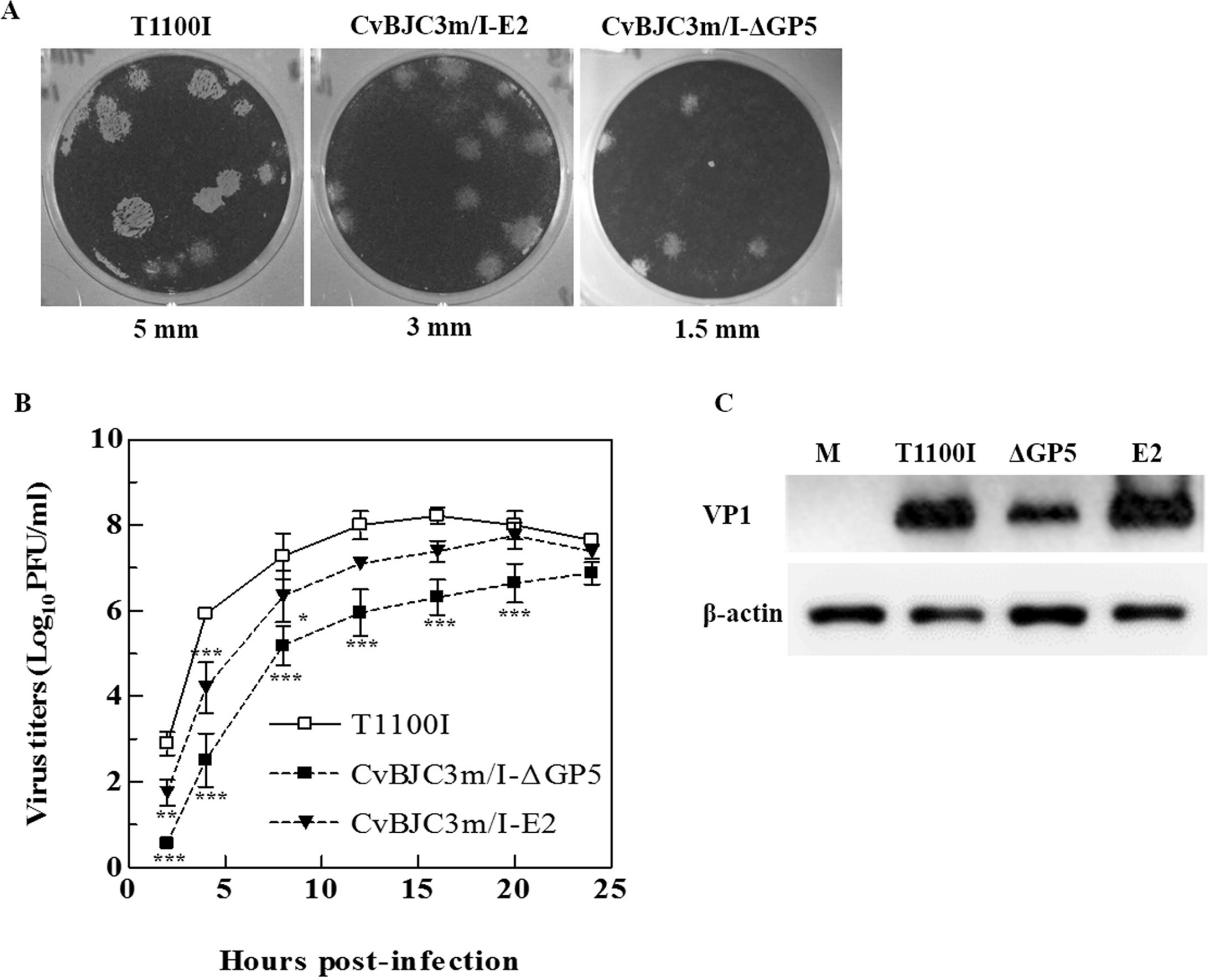
Replication of parental and recombinant viruses in BHK-21 cells. (A) Plaque morphology of recombinant viruses. (B) One-step growth curves of recombinant viruses. BHK-21 cells were infected with parental T1100I or recombinant viruses CvBJC3m/I-ΔGP5 and CvBJC3m/I-E2 at MOI 5. Freeze-thawed samples collected from duplicate wells at indicated time points (hpi) and virus titers determined using plaque assay. SD bars are shown for triplicate independent experiments. Asterisks represent statistically significant differences between parental T1100I and respective recombinant viruses as described in Material and Methods. (C) VP1 expression of recombinant viruses by western blot. Infected cells from the same cultures used for panel B were lysed and capsid protein VP1 was detected using anti-VP1 monoclonal antibody. M refers to mock infected BHK-21 cell lysates. ΔGP5 and E2 are herein abbreviated for CvBJC3m/I-ΔGP5 and CvBJC3m/I-E2 infected cell lysates respectively.

The recombinant viruses were titrated by a standard plaque assay as previously described [20]. To evaluate the inserted genes stability, the recombinant viruses were passed on BHK-21 cells for 4 additional passages and the insertions were sequenced using reverse-transcription PCR (RT-PCR) with the primers shown in S1 Table. For each passage, BHK-21 cell cultures were harvested at 24 h post-infection as mentioned above and titrated by plaque assay to determine the viral yields. To determine the plaque morphology of recombinant viruses, 10 plaques were randomly selected from duplicate wells and their sizes measured with a ruler. These measurements are reported as mean diameters in Table 1. The representative plaque images of parental and recombinant viruses are displayed in Fig 2A.

**Table 1:** Growth characteristics of the recombinant viruses in BHK-21 cells.

Rescued virus	Plaque size (mm)^a^	Virus titer (PFU/ml)^b^
T1100I	3.9 ± 0.4	1.7×10^8^
CvBJC3m/I-ΔGP5	1.2 ± 0.4*	6.3×10^6^
CvBJC3m/I-E2	2.1 ± 0.5	5.7×10^7^

^a^ Plaque size was expressed as mean diameters ± SD. The asterisk represents statistically significant differences between parental T1100I and CvBJC3m/I-ΔGP5.

^b^ Virus titer was determined as mean titer of three continuous passages in BHK-21 cells (P3 to P5).

### Growth curves of recombinant viruses in BHK-21 cells

The growth kinetics of the recombinant viruses were analyzed on the BHK-21 cell monolayers maintained in 96-well culture plates as previously described [20]. To quantitate the infection for single-step growth assays, BHK-21 cell monolayers were infected at a multiplicity of infection (MOI) of 5 PFU per cell and incubated at 37°C for 1h. Subsequently, the monolayers were washed three times with phosphate-buffered saline (PBS) to remove the unattached particles. 100μl of complete Dulbecco’s modified Eagle medium (DMEM) supplemented with 2% fetal bovine serum was added to each well. Duplicate wells were harvested at each time point, freeze-thawed for three times. Total virus yields were determined by plaque assay. Data for the growth kinetics for each virus represents three individual experiments.

### Indirect immunofluorescence assay and western blot detection of the expressed fusion proteins

The fusion proteins were detected by an indirect immunofluorescence assay (IFA) as previously described [21]. Briefly, BHK-21 monolayers infected with recombinant viruses were fixed with pre-chilled ethanol and respectively incubated with porcine polyclonal serum against GP5 glycoprotein (1:80) or E2-specific monoclonal antibody (1:400, kindly provided by Dr. Wang Qin, China Control Institute for Veterinary Drugs, Beijing, China). After interacting with FITC-conjugated second antibody, the infected cells were visualized for specific green fluorescence.

BHK-21 cells were infected with either parental T1100I, recombinant CvBJC3m/I-ΔGP5, or recombinant CvBJC3m/I-E2 viruses at an MOI = 5. Cell lysates were harvested at 16 hours post-infection (hpi) and analyzed by western blotting as previously described [22]. In brief, infected cells were washed and lysed in lysis buffer (20 mM Tris/HCl, pH 7.6, 150 mM NaCl, 1% Nonidet P-40, 0.5% sodium deoxycholate, 0.1% SDS). The lysates were then separated on a 12% SDS-polyacrylamide gel and transferred to a polyvinylidene difluoride (PVDF) membrane in a semi-dry transfer system. The membranes were blocked with 5% skimmed milk (in PBS, pH 7.2) overnight at 4°C and probed with respective antibodies. The membranes were incubated with primary antibodies at 37°C for 1h. Three primary antibodies were used in the western blot: anti-E2 monoclonal antibody (1:200, mouse), anti-VP1 monoclonal antibody (1:600, mouse, prepared and stored in the laboratory) and anti-GP5 porcine polyclonal serum (1:20). Subsequently, the membranes were incubated with an HRP conjugated goat-anti-mouse second antibody (1:10,000, Santa Cruz, CA) or HRP-goat-anti-porcine second antibody (1:15,000, Sigma-Aldrich, St. Louis, USA) at 37°C for 45 min and the bands were detected using ECL chemiluminescence and Amersham Hyper-Maxfilms, as recommended by the manufacturer (Amersham).

### Measurement of neutralization antibodies induced by recombinant viruses

Eight-week-old BALB/c mice were intraperitoneally immunized with 5×10^4^ PFU of either recombinant viruses or parental virus T1100I in DMEM containing 2% FBS and boosted with the same dose at 14 days post immunization (dpi). We collected the serum samples at the indicated time points by submandibular bleeding and measured the neutralizing antibodies against the respective pathogens with neutralization assays. For the PRRSV-specific neutralization assay, two-fold serial dilutions of serum (heat inactivated at 56°C for 30 min) from vaccinated or control animals were incubated with 100 TCID_50_ units of PRRSV JXwn06 strain at 37°C for 1h. This virus-serum mixture was added to MARC-145 cell monolayers in 96-well plates, incubated at 37°C for 5 days and monitored for virus-specific cytopathic effect (CPE). The neutralization endpoint was defined as the highest serum dilution in which 50% or less wells had obvious specific CPE.

CSFV C-strain was used in the test for the detection of antiviral neutralization antibodies. 200 TCID_50_ units of CSFV C-strain were incubated with serially diluted immunized or control serum samples (inactivated) at 37°C for 1h. PK-15 monolayers in 96-well plates were inoculated with the mixture and incubated at 37°C for 4 days. PK-15 monolayers fixed by pre-chilled ethanol were immunostained with the monoclonal antibody against E2 as the primary antibody and FITC-conjugated goat anti-mouse IgG as the second antibody (Sigma-Aldrich, St. Louis, MO). GFP positive cells were observed using a fluorescence microscope (Olympus, U-LH 50HG, Japan). The reciprocal of the highest dilution of serum samples at which the fluorescence was 50% or less than that of the control wells was taken as the serum neutralization titer.

### Statistical Analyses

All data were analyzed using GraphPad Prism software. Error bars denote standard deviation of the mean (SD) in all figures and the *P* values were determined using two-tailed student’s t-tests (*P < 0.05, **P < 0.01, ***P < 0.001).

## Results

### PRRSV ORF5 is partially deleted whereas CSFV E2 gene fragment is genetically stable in recombinant viruses

The ORF5 gene and A1A2 fragment in E2 gene were inserted at the nucleotide position 748 of T1100I genome according to the EMCV strain BJC3 sequence (GenBank accession no. DQ464062). Thus, the resulting viruses had the respective encoded foreign proteins fused between amino acid 5 and 6 of the L protein of EMCV T1100I (Fig 1). BHK-21 cells transfected with *in vitro* transcripts derived from linearized recombinant full-length cDNA clones pWSKBJC3m/I-GP5 and pWSKBJC3m/I-E2 had obvious CPE at 48 h and 36 h post transfection, as compared with 20 h of the cells transfected with mRNA from pWSKBJC3m/I. Sequence analysis of the corresponding insert regions in the viable viruses showed that the A1A2 gene in CvBJC3m/I-E2 was intact. However, the ORF5 gene carried by CvBJC3m/I-ΔGP5 had a 69nt deletion at its 3’ end along with a 48nt deficiency at the 5’ end of the adjacent EMCV L gene. The integrated A1A2 gene and partially deleted ORF5 gene were stably retained in the chimeric genomes for at least 4 passages when propagated in BHK-21 cells.

### Insertions into the leader protein-coding region of GP5 and E2 significantly affect EMCV replication

To further confirm whether heterologous gene insertions affect EMCV viral replication or not, we performed a set of comparative analyses of *in vitro* replication between parental and recombinant viruses. Plaque morphology analysis of the recombinant viruses, CvBJC3m/I-ΔGP5 and CvBJC3m/I-E2 suggested that their plaque size (Table 1) was approximately 30% and 60% as that of parental T1100I (Fig 2A). The growth kinetics indicated that CvBJC3m/I-E2 and CvBJC3m/I-ΔGP5 had delayed peaks of progeny titers at 20 hpi and 24 hpi,which were almost 0.8 and 2 log less than that of parental T1100I, respectively (Fig 2B). Although overall growth tendencies were similar with T1100I, the titers of CvBJC3m/I-ΔGP5 were remarkably reduced (*P* < 0.001) at all time points except 24 hpi and CvBJC3m/I-E2 exhibited less effective replication at 2 hpi (*P* < 0.01), 4 hpi (*P* < 0.001) and 8 hpi (*P* < 0.05), indicating that the engineered leader insertions had a significantly negative impact on EMCV growth. Consistent with the one-step growth kinetics, we indeed noted that recombinant virus CvBJC3m/I-ΔGP5 capsid protein VP1 level was apparently lower compared to parental virus T1100I at 16 hpi, whereas the other recombinant virus CvBJC3m/I-E2 produced comparable level of VP1 protein with parental T1100I at this time point (Fig 2C).

### Recombinant viruses express foreign polypeptides *in vitro*

**Fig 3:**
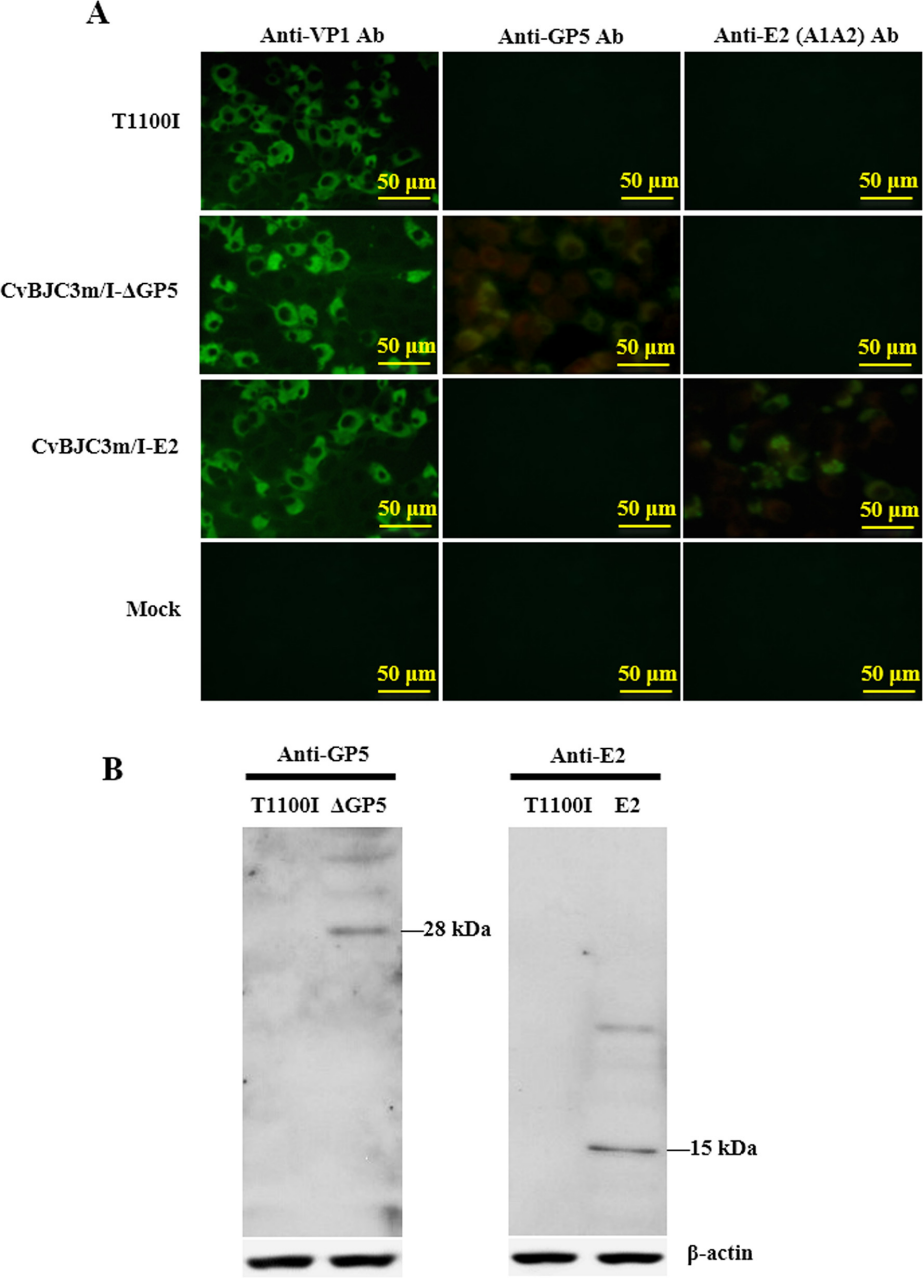
Determination of expressed fusion-proteins by IFA and western blot analysis. (A) IFA identification of fusion proteins. BHK-21 cells were infected with recombinant viruses or parental T1100I at MOI 5 and stained with anti-VP1, anti-GP5 or anti-E2 antibody at 16 hpi. The scale bars in the pictures represent 50 μm. (B) Western blot of fusion proteins. BHK-21 cell lysates harvested at 16 hpi were probed for fusion proteins with same antibodies. M refers to mock infected BHK-21 cell lysates. ΔGP5 and E2 are herein abbreviated for CvBJC3m/I-ΔGP5 and CvBJC3m/I-E2 infected cell lysates respectively.

To determine whether the recombinant viruses expressed the heterologous polypeptides, we infected BHK-21 cells with either parental or recombinant viruses. As a result, the fusion proteins L-ΔGP5 and L-E2 could be recognized by respective antibodies in BHK-21 cells infected with CvBJC3m/I-ΔGP5 and CvBJC3m/I-E2 whilst no signal was detected in mock- or T1100I-infected cells in IFA (Fig 3A). Western blot detection showed that the recombinant virus CvBJC3m/I-E2 produced a fusion protein of approximately 15 kDa and CvBJC3m/I-ΔGP5 expressed a protein of about 28 kDa (Fig 3B).

### Immunization of mice with recombinant viruses elicits neutralization antibodies against the respective pathogens from which the foreign sequences were derived

To further determine the immunogenicity of the two recombinant EMC viruses *in vivo*, we next immunized BALB/c mice with 5×10^4^ PFU of either recombinant viruses or parental virus and evaluated PRRSV- or CSFV-specific neutralization antibody level at 14, 28, 42 and 56 days after primary immunization. As shown in Table 2, anti-CSFV neutralization antibodies were detected in serum samples collected from mice inoculated with CvBJC3m/I-E2 but not from mice immunized with T1100I or 2% DMEM. Antibody titers increased to a peak of 1:36.8 at 42 days or 28 days after boosting with the same dose of recombinant virus and remained for another two weeks. Similar results were obtained in mice immunized with CvBJC3m/I-ΔGP5. Neutralization antibody titers doubled after the second immunization and peaked to 1:18.4 in two weeks with a slight decrease at 56 dpi.

**Table 2:** Neutralizing antibodies induced by recombinant EMC viruses in mice.

		Neutralization antibody titer				
Immunogen^a^	Antigen	0 days^d^	14 days^e^	28 days	42 days	56 days
T1100I		< 2	< 2	< 2	< 2	< 2
CvBJC3m/I-ΔGP5	PRRSV^b^	< 2	5.28	12.1	18.4	17.7
CvBJC3m/I-E2		< 2	< 2	< 2	< 2	< 2
2% DMEM		< 2	< 2	< 2	< 2	< 2
T1100I		< 2	< 2	< 2	< 2	< 2
CvBJC3m/I-ΔGP5	CSFV^c^	< 2	< 2	< 2	< 2	< 2
CvBJC3m/I-E2		< 2	13.9	27.9	36.8	28.5
2% DMEM		< 2	< 2	< 2	< 2	< 2

^a^ Eight-week-old male BALB/c mice (n = 5) were intraperitoneally immunized with 5×10^4^ PFU recombinant virus and T1100I in 0.2 ml 2% DMEM. Mice (n = 3) inoculated with 0.2 ml 2% DMEM in the same route were used as negative control.

^b^ Neutralization antibody titers were defined as the reciprocal of highest serum dilution that neutralized 100 TCID_50_ of JXwn06 and were expressed as the geometric mean value of 5 mice.

^c^ Neutralization antibody titers were defined as the reciprocal of highest serum dilution that neutralized 200 TCID_50_ of CSFV C-strain and were expressed as the geometric mean value of 5 mice.

^d^ Serum samples were collected at day 0 before immunization.

^e^ Mice were boosted with identical dose (5×10^4^ PFU) of either viruses or medium at 14 dpi.

## Discussion

In the present study, we fused foreign proteins between amino acid 5 and 6 in the leader protein of parental virus T1100I and generated two recombinant viruses. The resulting recombinant viruses, CvBJC3m/I-ΔGP5 and CvBJC3m/I-E2, generated smaller plaque size on BHK-21 cells (Fig 2A), suggesting an effect on viral replication and spread. Delayed peaks of replication and reduced viral yields were also observed for recombinant viruses in growth kinetics (Fig 2B), which indicated that heterologous genetic insertions curbed virus replication and the impairment had positive correlation with the size of inserts. This data was also consistent with the observations that the recombinant viruses had delayed occurrence of CPE after mRNA transfection, as well as less capsid protein (VP1) production at 16 hpi detectable by western blotting when compared to the parental virus (Fig 2C). One feasible explanation is that the recombinant viruses may package less efficiently than the parental virus due to the fact that foreign genes increase the length of the vector’s genome. It has been confirmed that extending the length of genome will result in negative consequences to virus packaging in the case of Mengo virus and TMEV [10, 23]. Given the fact that the insertions reduce the peak viral yields by 0.8 to 2 logs, it suggests that leader protein integrity is required for the overall replication of EMCV. Recombinant virus CvBJC3m/I-ΔGP5 had a 117nt deletion including a 69nt truncation at 3’ end of PRRSV ORF5 and a 48nt deficiency at 5’ end of EMCV leader gene at P0, however, no subsequent deletion was observed in the following passages to P5. This result is similar to Hammoumi group’s previous work, in which they reported that an EGFP insertion between amino acid 45 and 46 of leader protein resulted in 348bp or 534bp deletions of the EGFP gene fragment which resulted in decreased fluorescence *in vitro*. Another study using Mengo virus, which is genetically close to EMCV, suggested that insertion of gene fragments greater than 500 to 600bp would cause genetic deletions during passaging [24]. Thus, it is not surprising that we observed deletions in the PRRSV ORF5 (604bp) while the CSFV E2 gene (204bp) remained intact during virus rescue. However, truncated ORF5 maintained its stability during serial passages until P5, which is different from the continuous increase of spliced RNA genome in Hammoumi’s EGFP insertion. This may be explained by the fact that i) our PRRSV ORF5 is smaller in size than EGFP (717bp), and ii) ORF5 has a less G/C content (50.4%) than EGFP (62%), which was reported to be correlated with the insert stability [25]. The actual mechanisms that are involved in the deletion of inserted gene fragments remain unclear. To increase the tolerance of carrying heterologous genes by the live virus vector, Andino et al managed to place the foreign proteins with a recognition site for 3C^pro^ at the junction to the N-terminus of the poliovirus polyprotein precursor. Using this method, they have shown that poliovirus is capable of stably carrying insertions representing 15% (~1,110bp) of the viral genome size by releasing the foreign insertions via 3C^pro^ cleavage [2].

As described previously, the threonine substitutions at amino acid position 100 of VP1 remarkably lower the neuropathogenicity of EMCV. Furthermore, the recombinant viruses, based on its already attenuated parental virus, had further increased the LD_50_ units to as high as 3×10^6^ PFU. This may be due to less efficient replication of the recombinant viruses in the infected mice. However, an impaired role of the L protein in interfering host anti-viral responses resulting from gene insertions might be a more convincing explanation. Cardiovirus L protein is a primary viral determinant of pathogenesis and collective data has shown that it can antagonize type I interferon signaling and interfere with nucleocytoplasmic trafficking of host proteins [26–28]. Indeed, our data suggest a better insertion point between amino acid 5 and 6 rather than amino acid 45 and 46 in Hammoumi’s work, since the resultant EGFP recombinant virus remained pathogenic in mice [11]. In our hands, vector attenuation results in the usage of a higher dose to animal immunization, which permits studies on immunogenicity of the fusion proteins. However, we cannot rule out the possibility that our recombinant viruses could revert back to a virulent form during *in vivo* infection. As shown in Table 2, 5×10^4^ PFU immunization was able to induce antibody responses against the foreign proteins and the second immunization at 14 days post the initial immunization enhanced the responses, which suggests that the fusion proteins are immunogenic *in vivo*. Although GP5 fusion protein had a 23-amino-acid linear epitope deletion at the C-terminus, CvBJC3m/I-ΔGP5 still maintained the neutralization epitopes located in the middle of GP5 ectodomain [29] and is able to induce mild neutralizing antibodies in mice. This result is not surprising since it has been reported that virus-like particles composed of PRRSV GP5 and M protein were capable of eliciting neutralizing antibodies during mice immunization [30]. However, whether the phenotype observed in our mice model would be able to translate into porcine vaccine development is still questionable as PRRSV GP5-based vaccines only induce unsatisfactory neutralization antibodies and confer limited protection upon challenge [31–33]. Similarly, although the E2 recombinant virus successfully induces neutralizing antibodies against the CSFV C-strain in our mouse system, further investigations have to be performed to evaluate its protective immune response induction in a pig model.

In conclusion, our findings demonstrate that an attenuated EMCV could serve as a vector to express heterologous antigens, which provides a useful tool to study the immunogenicity of the heterologous proteins in a mouse model. The lab is now interested in studying the protective immune responses of EMCV recombinant viruses in pigs. These studies may potentially inform us whether EMCV could be engineered into live vaccines to protect pigs from various pathogens infection.

## Supporting Information

S1 Table Primers used in the study. (PDF)
