# Supplementary material for: Recombinant Encephalomyocarditis Viruses Elicit Neutralizing Antibodies against PRRSV and CSFV in Mice
Source: PLoS One. 2015 Jun 15;10(6):e0129729. doi: 10.1371/journal.pone.0129729 (PMC4468123; doi:10.1371/journal.pone.0129729)
Supplement: S1 Table — (PDF) [file pone.0129729.s001.pdf]

S1 Table. Primers used in the study.

Primer[a]	Template[b]	Sequence (5’- 3’)[c]	Use
EMLP-F	EMCV genome	GCCCTGTCTTCTTGACGAGC	Left arm amplification
LP5-GP5-R	EMCV genome	***GTCAAGCACTTCCCCAACAT***CATGGTTGTGGCCATATTATCAT	Left arm amplification
fGP5-F	JXwn06-ORF5	***ATGATAATATGGCCACAACCATG***ATGTTGGGGAAGTGCTTGAC	ORF5 amplification
fGP5-R	JXwn06-ORF5	***CGCAAGTCTCTTGTTC***CTAGAGACGACCCCATTGTTC	ORF5 amplification
LP6-GP5-F	EMCV genome	***GAACAATGGGGTCGTCTCTAG***GAACAAGAGACTTGCG	Right arm amplification
1B-R	EMCV genome	GCACCATAACCGACAAGACG	Right arm amplification
EMLP-F	EMCV genome	GCCCTGTCTTCTTGACGAGC	Left arm amplification
LP5-E2-R	EMCV genome	***CAGGACTCGTATCAAACGGGCACAG***CATGGTTGTGGCCATATTATCAT	Left arm amplification
fA1A2-F	$CSFV_{SM}$ E2-A1A2	***ATGATAATATGGCCACAACCATG***CTGTGCCCGTTTGATACGAGTCCTG	E2 amplification
fA1A2-R	$CSFV_{SM}$ E2-A1A2	***CGCAAGTCTCTTGTTC***GGTCACACAATCCATTCTGTGTGGGA	E2 amplification
LP6-E2-F	EMCV genome	***TCCCACACAGAATGGATTGTGTGACC***GAACAAGAGACTTGCG	Right arm amplification
1B-R	EMCV genome	GCACCATAACCGACAAGACG	Right arm amplification

[a] F refers the forward PCR primer and R stands for the reverse PCR primer.

[b]Specific gene fragments were amplified from templates including EMCV BJC3 (GenBank accession no. DQ464062), PRRSV JXwn06 (GenBank accession no. EF641008) and CSFV Shimen/HVRI (GenBank accession no. AY775178).

[c] 5’ overhangs are shown in boldface italics.
